# Primary retroperitoneal germ cell tumor in an adult female: A case report

**DOI:** 10.1097/MD.0000000000019170

**Published:** 2020-02-14

**Authors:** Qitian Lou, Weigen Wang, Wenjie Liang

**Affiliations:** a Department of Radiology, Ninghai First Hospital, Ninghai, Ningbo, Zhejiang; b Department of Radiology, The First Affiliated Hospital, College of Medicine, Zhejiang University, Hangzhou, Zhejiang, China.

**Keywords:** CT, extragonadal germ cell tumor, imaging, MRI

## Abstract

**Rationale:**

Primary retroperitoneal germ cell tumors are uncommon and especially rare in female patients. However, this type should be included in the differential diagnosis of retroperitoneal tumors that may metastasize from the gonads and be a primary tumor.

**Patient concerns:**

An abdominal mass was detected in a 38-year-old woman during physical examination, which was accompanied by left renal obstructive hydrops. She was admitted to our institution for further investigation. The patient had no obvious clinical symptoms, and the levels of serum tumor markers did not signifificantly increase. Abdominal noncontrast enhanced and contrast-enhanced computed tomography revealed a retroperitoneal neoplasm that invaded the left ureter, thereby causing left hydronephrosis.

**Diagnoses:**

Imaging examination characterized the tumor as malignant based on its invasion in the left ureter. Histopathology and immunohistochemistry confifirmed the resected tumor as a dysgerminoma. The primary gonad-derived germ cell tumor was not found in the pelvis; therefore, the patient was fifinally diagnosed with primary retroperitoneal germ cell tumor.

**Interventions:**

Preoperative examination was completed, and the retroperitoneal mass was resected.

**Outcomes:**

During the short-term follow-up, no tumor recurrence was detected.

**Lessons:**

Primary retroperitoneal seminoma should be included in the differential diagnosis of primary retroperitoneal tumors in female patients. The primary retroperitoneal seminoma/anaplastic tumor has an obvious occupying effect and can easily invade the surrounding structures. However, surgical resection of such tumors is an optional treatment strategy.

## 1 Introduction

Germ cell tumors are caused by the differentiation of primordial germ cells or pluripotent blasts occurring in or outside the gonads. Such tumors mainly occur in the gonads, including the testes and ovaries. Extragonadal germ cell tumors outside the gonads account for only 1% to 2.5% of germ cell tumors. They can occur outside the gonads around the body midline (eg, mediastinum, posterior peritoneum, pineal region, and appendix) mainly due to the incomplete migration of primordial germ cells during embryonic development.^[1]^ Although the posterior peritoneum is the second most common site for extragonadal germ cell tumors, these tumors account for only 5% of primary retroperitoneal tumors.^[2]^ Primary retroperitoneal germ cell tumors almost exclusively affect men. Herein, we report a case of primary retroperitoneal germ cell tumor in a woman along with detailed clinical imaging and literature review.

## 2 Case report

An abdominal mass was detected through ultrasonography in a 38-year-old woman during local physical examination 2 days earlier and was referred to a local hospital for further examination. Abdominal computed tomography (CT) showed a mass in the abdomen, with slightly dilated left upper ureter along with mild hydronephrosis, probably due to mass compression. The patient did not report abdominal pain or distension, nausea or vomiting, and diarrhea or melena, and was admitted to our institution for further examination and treatment. The patient was generally in good health, with stable vital signs and no recent significant change in weight. Physical examination indicated a soft abdomen without tenderness or rebound tenderness. The patient had history of hypertension (>5 years) and was treated with oral amlodipine tablet (1 q.i.d) and irbesartan capsule (1 q.i.d), with reasonably well-controlled blood pressure.

**Figure 1:**
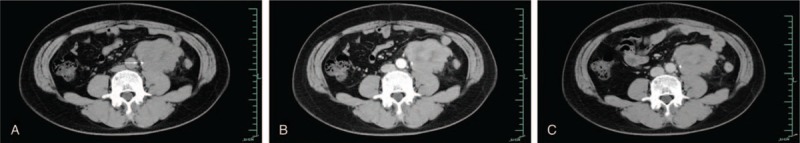
(A) Abdominal unenhanced CT showed an irregularly shaped retroperitoneal soft tissue mass with heterogeneous density and CT value of ∼47.5 HU. The tumor surrounding the left ureter, and the indwelling tube is observed in the ureter (arrow). Contrast-enhanced CT showed heterogeneous and moderate enhancement of the mass, surrounding the left ureter. The arterial phase value (B) of the tumor on CT was ∼61.6 HU, while the venous phase value (C) on CT was ∼63.5 HU. CT= computed tomography.

After admission, routine laboratory blood testing did not show significant abnormal changes. Liver function and renal function were unremarkable, except a slight increase in total cholesterol (7.14 mmol/L, normal range: 3.14–5.86 mmol/L). The levels of serum tumor markers, including alpha fetoprotein (AFP), carcinoembryonic antigen, carbohydrate antigen 125 (CA125), and CA199 did not show a significant increase. Abdominal unenhanced and contrast-enhanced CT at our institution revealed the presence of a heterogeneous mass in the left retroperitoneal region, suggestive of a malignant tumor (Fig. 1). The tumor affected the left ureter leading to left hydronephrosis. After preoperative preparation, a massive retroperitoneal tumor was resected. During the operation, we did not find obvious ascites in the abdominal cavity or evident nodules in the abdominal wall, pelvic wall, liver, gallbladder, spleen, pancreas, or omentum. The left retroperitoneal tumor was firm with unclear boundaries and invaded the left colon, partial mesentery, and bordered the left ureter. With reference to the clinical stage of the retroperitoneal tumor, the preoperative clinical stage of our case is stage Ib (T2N0M0).

**Figure 2:**
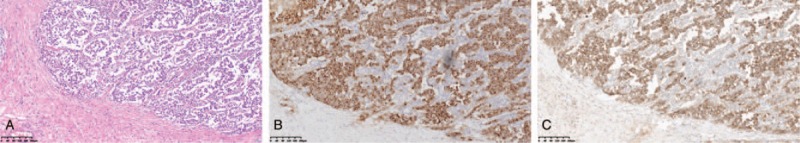
(A) Tumor cells were oval, eosinophilic, polymorphonuclear, showed atypia and were arranged in a slab-like pattern with lymphocyte infiltration (HE, ×100). IHC indicated PLAP+ (B) and CD117+ (C) in tumor mass (×100). HE = hematoxylin and eosin, IHC = immunohistochemical.

After resection, the large peritoneal mass measuring ∼10 × 6.5 × 5 cm^3^ was subjected to pathological examination. Microscopic examination showed that the tumor cells were oval with acidophilus in the cytoplasm and had visible vacuoles. The cells showed a nest-like and slab-like pattern arrangement with invasive growth and atypia. They contained polymorphonuclear cells and obvious nucleoli accompanied by lymphocytes and minor plasma cell infiltration. The tumor cells were rich in internal sinusoids and visible mitosis. In addition, the tumor surrounded the ureter and descending colon, without evidence of metastasis in the peritumor or mesenteric lymph nodes. Immunohistochemical results were as follows: pan-cytokeratin (−), vimentin (−), octamer-binding transcription factor 4 (+), placental alkaline phosphatase (+), CD30 (−), CD117 (+), AFP (−), D2-40 (+), beta-human chorionic gonadotropin (β-hCG) (−), glypican-3 (−), CD3 (lymphocyte +), CD20 (lymphocyte +), anaplastic lymphoma kinase (−), CD138 (plasma cell +), HMB45 (−), MelanA (−), S-100 (−), CgA (−), and synapsin (−). The pathological diagnosis was retroperitoneal giant germ cell tumor, whose morphology and immunophenotype were consistent with dysgerminoma (Fig. 2). The patient recovered well after surgery and did not show obvious signs of recurrence during the short-term follow-up of ∼3 months.

## 3 Discussion

Most cases of extragonadal germ cell tumors occur in men. The age of onset of seminoma is 23 to 70 years; however, occurrence is most common in the fourth decade of life.^[3]^ Primary retroperitoneal extragonadal germ cell tumors should be excluded from retroperitoneal metastasis of gonadal germ cell tumors. This is because 30% to 50% of testicular gonadal tumors can give rise to retroperitoneal metastasis, accounting for the majority of retroperitoneal germ cell tumors.^[2]^ However, unlike previous cases, the present case was a female. Moreover, because there was no primary lesion in the pelvic cavity, our case could be excluded from metastatic tumor cell tumors. Currently, there is debate among researchers regarding the existence of this disease. However, our case supports the independent existence of primary retroperitoneal germ cell tumors, because most retroperitoneal germ cell tumors are accompanied with testicular lesions.^[4]^ Some common clinical manifestations of retroperitoneal germ cell tumors are abdominal and back pain,^[3]^ occasionally also including testicular pain, bloating, lower extremity edema, and obstructive nephropathy.^[5–10]^ In this case, the mass was accidently detected through physical examination without clinical symptoms. Thus far, only 1 other case has been diagnosed during physical examination in a previous study.^[11]^ Based on the different classifications of germ cell tumors, patients with retroperitoneal germ cell tumors exhibit different abnormal results in laboratory examinations.^[1,6,7]^ For instance, yolk sac tumors, choriocarcinomas, and retroperitoneal seminomas may be accompanied by abnormal elevation of serum AFP,^[6,7]^ hCG,^[1]^ and hCG/lactate dehydrogenase ratio, respectively.^[3,9]^ However, the absence of an increase in serum tumor markers cannot rule out the possibility of retroperitoneal germ cell tumors.

Primary retroperitoneal primary germ cell tumors do not present specific imaging characteristics.^[1]^ Retroperitoneal seminoma can be ultrasonically shown as a hypoechoic mass.^[11]^ Primary retroperitoneal seminoma can be shown as an isolated retroperitoneal mass on CT, measuring 1 to 30 cm, with uniform density, regular or irregular margins, and clear boundaries.^[1,2,5,9,11]^ Tumors can easily surround and further invade blood vessels (eg, vena cava, abdominal aorta, iliac vessels) and invade the ureter.^[5,9,11]^ Through contrast-enhanced scanning, primary retroperitoneal seminoma can be characterized based on mild-to-moderate uniformity or uneven enhancement,^[5,9,11]^ with segregated or punctate calcification in lesions.^[1]^ In magnetic resonance imaging, primary retroperitoneal seminoma shows a low-to-medium signal in T1-weighted imaging (T1WI), and a medium-high signal relative to the muscle signal in T2WI.^[12]^ The intratumoral separation exhibits a low signal in T2WI.^[1]^ In contrast, non-seminomatous germ cell tumors are more likely to manifest as massive inhomogeneous and highly invasive masses owing to the necrosis and hemorrhage within the lesion.^[1,2,6–8]^ Tumors containing teeth and mature fat are generally teratomas.^[2]^ In terms of differential diagnosis, metastatic retroperitoneal germ cell tumors should be distinguished from primary retroperitoneal germ cell tumors, especially in men.^[12]^ The midline position generally contributes to the diagnosis of primary retroperitoneal germ cell tumors rather than metastatic tumors.^[12]^ Biopsy and other methods can be used to determine whether the testis has primary lesions to confirm the diagnosis of primary retroperitoneal germ cell tumors. In our patient, the retroperitoneal germ cell tumor was finally diagnosed as a primary retroperitoneal lesion, because imaging did not reveal a primary neoplastic lesion in the accessories on either side. Additionally, primary retroperitoneal germ cell tumor should also be distinguished from other primary retroperitoneal tumors, such as lymphomas, neurogenic tumors, and sarcomas.

Primary retroperitoneal germ cell tumor should be diagnosed pathologically, and immunohistochemistry (IHC) plays a vital role in its diagnosis and differential diagnosis.^[13]^ Among the IHC indicators, the positive expression of placental alkaline phosphatase (PLAP), CD117, and Octamer-binding transcription factor 3/4 (OCT3/4) is considered to show important diagnostic value for the diagnosis of seminomas.^[13]^ Similarly, in our female case, all the above 3 IHC indicators were positively expressed, which were in consistent with the characteristics of dysgerminoma.

Currently, primary retroperitoneal seminoma is mainly treated by chemotherapy (including Cisplatin or Carboplatin), radiotherapy or radiochemotherapy.^[3]^ Meanwhile, the treatment options for retroperitoneal non-seminoma include conventional cisplatin-based chemotherapy and/or surgery as well as radiotherapy.^[3]^ About 1/2 primary retroperitoneal seminomas are metastatic, among which, abdomen and paratracheal lymph nodes have been identified as the most frequent sites of metastasis, accounting for 50% of metastatic retroperitoneal non-seminomas.^[3]^ Additionally, the rest 50% of metastatic retroperitoneal non-seminomas generally metastasize to lungs.^[3]^ Thus, the prognosis for primary retroperitoneal seminoma is superior to that of retroperitoneal non-seminoma. For our case, the relatively good prognosis was achieved following active surgical resection, which was equally worthwhile on the resectable primary peritoneal dysgerminoma.

Finally, primary retroperitoneal germ cell tumors are uncommon, which are especially rare in women, and should be differentiated from metastatic germ cell tumors and other primary retroperitoneal tumors. Imaging examinations contribute to the clinical grading of tumors, while surgical treatment should be preferentially considered for primary peritoneal dysgerminoma.

## Author contributions

**Conceptualization:** Wenjie Liang.

**Writing – original draft:** Qitian Lou, Weigen Wang, Wenjie Liang.

**Writing – review and editing:** Qitian Lou, Weigen Wang, Wenjie Liang.
